# Unexpected Movement of the Esophagus across the Aorta

**DOI:** 10.3390/diagnostics12071758

**Published:** 2022-07-20

**Authors:** Hyun Ho Choi, Soo-Yoon Sung, Yoon Ho Ko

**Affiliations:** 1 Department of Internal Medicine, Uijeongbu St. Mary’s Hospital, College of Medicine, The Catholic University of Korea, Seoul 11765, Korea; choihyunho21@hanmail.net; 2 Department of Radiation Oncology, Eunpyeong St. Mary’s Hospital, College of Medicine, The Catholic University of Korea, Seoul 03312, Korea; 3 Division of Oncology, Department of Internal Medicine, Eunpyeong St. Mary’s Hospital, College of Medicine, The Catholic University of Korea, Seoul 03312, Korea; koyoonho@catholic.ac.kr

**Keywords:** esophagus, movement, tumor regression

## Abstract

Tumor regression throughout treatment would induce organ movement, but little is known of this in the esophagus. To achieve successful tumor regression, radiation therapy requires several weeks of radiation to be delivered accurately to the tumor. Usually, a 5–10 mm margin is allowed for set-up error and internal organ motion. Our case exhibited an unexpectedly large movement of the esophagus across the aorta with tumor regression that extended outside the margin and thus outside the radiotherapy field. These movements may affect subsequent invasive procedures or treatment during cancer therapy. After the unexpected large movement of the esophagus due to tumor regression, we revised the radiotherapy plan to reflect the new esophageal position. This implied that regular imaging and close monitoring are required during treatment of esophageal cancer.

A 79-year-old man was diagnosed with esophageal cancer during an evaluation of dysphagia. Upper endoscopy revealed a lumen-encircling mass 32 cm from the incisors (Figure 1); a biopsy specimen revealed squamous cell carcinoma. Distal esophageal wall thickening and multiple metastatic lymph nodes were observed upon computed tomography (CT) of the chest and abdomen (Figure 2A,B). The esophageal cancer stage was T4N2M0.

Definitive concurrent chemoradiation was scheduled. The radiation dose was 50.4 Gy/28 fractions over 5.5 weeks. The patient had received continuous infusion of 5-fluorouracil (1000 mg/m^2^) on days 1 through 4 and days 22 through 25 and cisplatin (75 mg/m^2^) was given on days 1 and 22. By 3 weeks after commencement of chemoradiation, the esophagus was outside of the radiotherapy field during image-guided radiotherapy. Follow-up CT revealed that the esophagus had moved from the right of the aorta to the left (Figure 2C,D). Fusion of the radiation plan and follow-up CT showed that prescription dose did not cover the entire esophagus (Figure 3).

Rumor regression throughout treatment would induce organ movement [1,2,3]. To achieve successful tumor regression, radiation therapy requires several weeks of radiation to be delivered accurately to the tumor [4]. Usually, a 5–10 mm margin is allowed for set-up error and internal organ motion. However, this case exhibited an unexpectedly large movement of the esophagus across the aorta with tumor regression that extended outside the margin and thus outside the radiotherapy field. Though organ movement with tumor regression is usually shown in the uterus, rectum and lung, little known of this in the esophagus [2,5]. These movements may affect subsequent invasive examinations or treatment during cancer therapy. In this case of unexpected large movement of the esophagus due to tumor regression, we revised the radiotherapy plan to reflect the new esophageal position. This implied that regular imaging and close monitoring are required during the treatment of esophageal cancer, especially for large esophageal lesions. Metal markers or clips would be helpful in monitoring the movement of the esophagus because they can be localized on a plain radiograph.

## Figures and Tables

**Figure 1:**
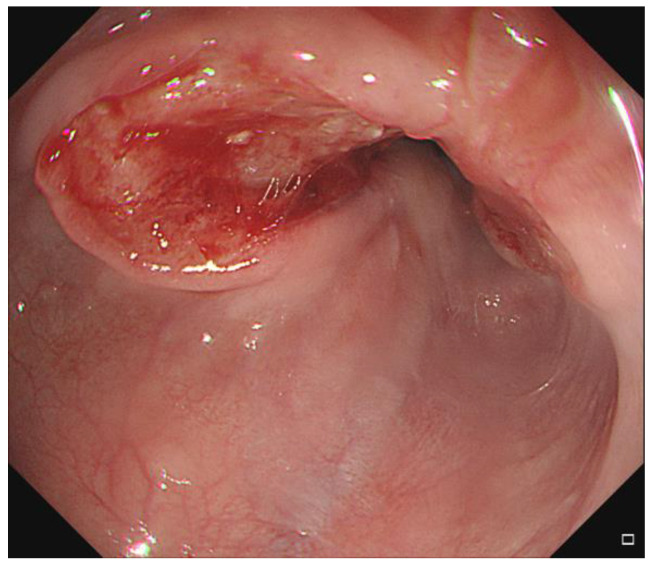
Endoscopic finding of the esophageal lesion.

**Figure 2:**
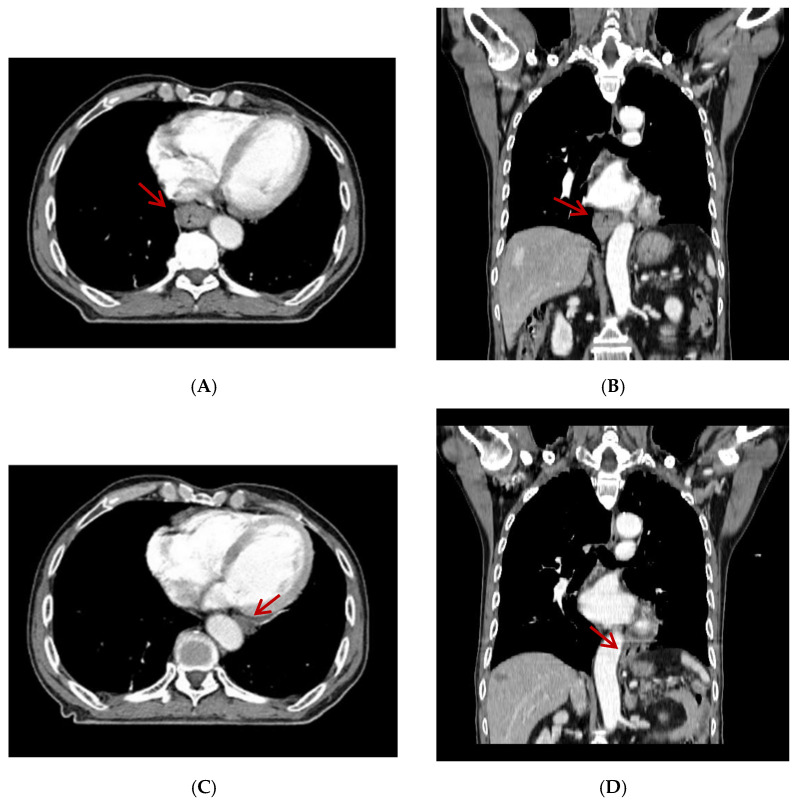
Distal esophageal wall thickening (arrow) was found on axial view (**A**) and coronal view (**B**) of initial computed tomography (CT) scan. Metastatic lymph nodes were not seen on these CT slices. Follow-up computed tomography revealed that the esophagus had moved from the right of the aorta to the left on axial view (**C**) and coronal view (**D**) (arrow).

**Figure 3:**
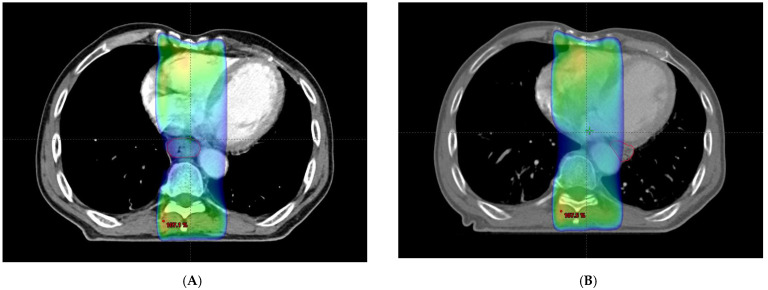
(**A**) Initial radiation plan. Esophagus (red line) is covered with 95% of prescription dose. (colored area). (**B**) Radiation plan with moved esophagus (red line). Part of esophagus was outside radiation field.

## Data Availability

The data presented in this study are available on request from the corresponding author.
